# Exploring the Broad Spectrum of Titanium–Niobium Implants and Hydroxyapatite Coatings—A Review

**DOI:** 10.3390/ma17246206

**Published:** 2024-12-19

**Authors:** Radu Radulescu, Marina Meleșcanu Imre, Alexandra Ripszky, Florentina Rus, Alexandra Popa, Mihai Moisa, Cristian Funieru, Razvan Ene, Silviu Pituru

**Affiliations:** 1 Department of Biochemistry, Faculty of Dental Medicine, University of Medicine and Pharmacy Carol Davila, 8 Eroilor Sanitari Blvd, 050474 Bucharest, Romania; radu.radulescu@umfcd.ro (R.R.); alexandra.ripszky@umfcd.ro (A.R.); florentina.rus-hrincu@umfcd.ro (F.R.); alexandra.popa@drd.umfcd.ro (A.P.); mihairadu.moisa@drd.umfcd.ro (M.M.); 2 Department of Complete Denture, Faculty of Dental Medicine, University of Medicine and Pharmacy Carol Davila, 17-23 Calea Plevnei, 010221 Bucharest, Romania; marina.imre@umfcd.ro; 3 The Interdisciplinary Center for Dental Research and Development, Faculty of Dental Medicine, “Carol Davila” University of Medicine and Pharmacy, 19-21 Jean Louis Calderon Street, 020021 Bucharest, Romania; silviu.pituru@umfcd.ro; 4 Department of Preventive Dentistry, Faculty of Dental Medicine, University of Medicine and Pharmacy Carol Davila, 4 Eforiei, 050037 Bucharest, Romania; 5 Orthopedics and Traumatology Department, “Carol Davila” University of Medicine and Pharmacy, 050474 Bucharest, Romania; 6 Department of Professional Organization and Medical Legislation-Malpractice, “Carol Davila” University of Medicine and Pharmacy, 020021 Bucharest, Romania

**Keywords:** titanium, niobium, titanium–niobium implants, biocompatibility, hydroxyapatite, coating, physical deposition, plasma spray, rf magnetron sputtering

## Abstract

Tooth loss replacement using dental implants is becoming more frequent. Traditional dental implant materials such as commercially pure titanium and titanium aluminum vanadium alloys have well-proven mechanical and biological properties. New titanium alloying metals such as niobium provide improved mechanical properties such as lower elastic modulus while displaying comparable or even better biocompatibility. Hydroxyapatite coatings are a well-documented and widely used method for enhancing dental implants’ surface characteristics and properties and could provide a useful tool for further enhancing titanium–niobium implant properties like osteointegration. Among several coating techniques, physical deposition methods and, in particular, vapour deposition ones are the most used due to their advantages compared to wet deposition techniques for hydroxyapatite coating of metallic surfaces like that of dental implants. Considering the scarcity of data concerning the in vivo evaluation of titanium–niobium biocompatibility and osteointegration and the lack of studies investigating coating these new proposed alloys with hydroxyapatite, this review aims to further knowledge on hydroxyapatite-coated titanium niobium alloys.

## 1. Introduction

Tooth loss is a prevalent health issue that can be caused by various factors such as complicated cavities, periodontitis and even trauma, impacting around 2.5 billion people, especially in disadvantaged and low-income populations [1]. As a solution, dental implants are commonly used to offer support for replacing missing teeth, and this practice has been in existence for a considerable period of time [2], with a long-term survival of well over 95% being reported [3]. 

The study of dental implant designs, materials, and techniques has experienced a significant increase in research activity in past years and is anticipated to grow further in the future [4]. After the first successful osteointegrated implant, the field experienced continuous improvements like computer-guided surgery, the development of immediate implant loading techniques leading to all-on-four or all-on-six prosthodontics, advanced surface treatments and digital dentistry integration [1].

Currently, approximately over 450,000 dental implants are inserted annually, with an estimated success rate of 95% for single-tooth replacement using an implant-supported crown, and it is regarded as a low-risk procedure [5].

Implant manufacturing materials must meet several requirements [6]:

- Be as light as possible while maintaining adequate mechanical strength (implants must be able to withstand a masticatory pressure sometimes exceeding 60 kgf/cm^2^);
- Ease of manufacturing and shape stability;
- Corrosion resistance on contact with saliva and blood;
- Lack of toxicity and carcinogenetic effects;
- Lack of galvanic interactions with other metals.

In 1981, Albrektsson was the first to propose the term “osteointegration” to describe the structural and functional connection between living bone and the surface of a load-carrying implant [7]. Osteointegration was defined as a direct structural and functional connection between bone and implant surface without the interposition of fibrous tissue in order to achieve long-term stability and functionality [8]. Optimal osseointegration before prosthetic loading is considered a prerequisite demand for long-term clinical success [7].

The rate of bone resorption around dental implants is the main measure for clinical success and is influenced by implant-dependent factors such as implant shape design and surface characteristics, the biocompatibility of implant material, implant storage conditions or surgical-dependent factors such as recipient bone quality, surgical technique and operator skill. These factors are critical for a low rate of bone loss and thus to long-term clinical success [9]. Biocompatibility is defined as the ability of a material to interact with the host’s fluids and tissues without eliciting harmful effects while fulfilling its desired function [10].

Implant shape, surface characteristics and bone quality are the key factors in the bone–implant interface [11], the critical area where adhesion between host tissue and implant surface occurs. Implant surface properties influence and regulate adjacent cellular activity both in the postsurgical healing phase and in the osseointegration one [12].

The leading causes for implant failure are bacterial infection and the consequent inflammatory response and can be either peri-implant mucositis or peri-implantitis [13].

Titanium and titanium alloy implants are considered the gold standard, offering survival rates of up to even 100% with marginal bone loss between 0.125 mm and 1.2 mm [14]. Hydroxyapatite-coated implants exhibit a bone loss of around 0.75 mm [15] while titanium zirconia implants display between 0.8 mm and 1 mm [16]. There are no long-term in vivo studies evaluating the success rate of titanium–niobium (Ti-Nb) implants even though they exhibit better mechanical properties than classical implants, which will be further discussed in this review that highlights in vitro studies showing promising results, driving the need for further studies.

Based on the response of the host, Sotova et al. divided materials used for dental implants into three categories: bioactive, bioinert or biotolerant. Biotolerant materials such as stainless steel or cobalt chromium induce osteogenesis through their irritative effect at the boundary between bone and implant surface, generating soft fibrous tissue separating the two [17].

Chemically inert materials such as titanium and titanium alloys, tantalum, alumina or zirconia induce contact osteogenesis with surrounding bone tissue due to the inert nature of their surfaces in favourable mechanical conditions [17]. Connective osteogenesis is induced by bioactive materials such as glass, glass ceramics or calcium phosphate ceramics through the presence of calcium and phosphate ions on the surface interacting with bone hydroxyapatite, generating a chemical connection [17].

The most used dental implants are titanium ones, either pure titanium (pTi) or titanium alloys with aluminum and vanadium [18]. Currently, pure titanium implants (pTi) come in four grades between I and IV and contain between 98% and 99.6% pure titanium. Grade IV is the most used out of the four grades because it contains the highest oxygen content of 0.4%, conveying the best mechanical strength among pTi implants [18]. The most used titanium alloys are Ti-Al-V, usually Ti-6Al-4V, which confer better mechanical, such as strength and ductility and modified corrosion resistance [19].

In order to enhance the properties or to improve the shortcomings of titanium implants, surface modifications can be made such as adding titanium nanostructures (nanotubes, nanopores, nanofibers, nanowires or silicate nanoparticles), mechanical modifications (polishing and surface roughing by blasting), chemical and electrochemical modifications (acid etching, oxidation and plasma electrolytic oxidation), coating the implant surface with bioactive molecules such as collagen, hydroxyapatite or growth factors or changing the alloying elements [19].

## 2. Niobium Titanium Alloys

Titanium alloys such as Ti-6Al-4V are used because of the favourable properties aluminum and vanadium confer such as low density, high strength and very good biocompatibility [20].

However, the main mechanical disadvantage is the high elastic modulus, which is significantly higher than that of the surrounding bone, leading to implant failure as a consequence of bone resorption [21]. Human bone has an elastic module value between 10 and 20 Gpa for cortical bone and 1.5 and 2.5 Gpa for cancellous bone compared to the average of 110 Gpa for titanium implants (105–110 GPa for cpTi and 110–120 GPa for Ti-6Al-4V) [22,23]. The Ti-Zr alloy elastic modulus is still very high, at an average of 80–110 GPa [24], while Ti-Nb displays a much lower value, resembling that of natural bone, which will be discussed further in this review. An overview of elastic modulus values can be observed in Table 1.

Dental implants, their surface in particular, come in contact with a series of chemical and physical factors such as friction, micro-movements, host inflammatory responses, bacterial metabolic products and chemical and electrochemical compounds from surrounding tissues [25].

The most used alloying metals added to titanium in order to enhance the alloy’s mechanical and biological properties, aluminum and vanadium, are increasingly linked with negative local effects such as bone fragility or inflammation and even negative systemic effects such as significant neurotoxicity associated with Alzheimer’s disease [26]. These ions are released from implant alloys through corrosion or are byproducts of surface blasting with aluminum oxide during surface processing [27].

Studies also linked vanadium and aluminum with cellular growth inhibition and oxidative stress promotion [28]. Aluminum inhibits mitochondrial processes by competing with Fe^3+^ [29], and Kermani et al. documented TAU protein inhibition by aluminum oxide, leading to structural damage and apoptosis [30]. Higher levels of Al^3+^ ions were found in cases of peri-implantitis induced by the more corrosive local environment compared to that found in healthy patients [31,32,33].

The main focus of reducing Young’s modulus in titanium alloys is finding suitable replacing metals for aluminum (Al) and vanadium (V) substitution. The elastic modulus or Young’s modulus measures the stiffness degree of a material taking into consideration the ratio of stress compared to the degree of strain or deformation [34]. 

Based on their crystalline structure, titanium alloys can be categorized as α phase alloys, near α alloys, α + β alloys and β phase alloys. The α phase has a closed-packed hexagonal spatial structure and the β phase has a body-centered cubic arrangement; the α phase can allotropically transform into the β phase according to the temperature, with 882 °C being the temperature threshold between these phases [35].

α phase titanium has lower mechanical properties and is not used in implant manufacturing, with the main stabilizing elements being Al, zirconium (Zr), O, N and C; one of their effects is increasing the α to β transformation temperature [36]. They display good mechanical resistance, high ductility and good corrosion resistance but suffer from high stiffness, leading to stress shielding and a mismatch in stiffness that impacts how mechanical forces are transmitted to the surrounding bone, leading to bone resorption [23].

β phase stabilizers can be divided based on the addition of alloying elements into isomorphous elements such as Nb (Niobium), V, molybdenum (Mo) and tantalum (Ta) and eutectoid ones such as manganese (Mn), chromium (Cr), silicon (Si), iron (Fe), cobalt (Co), nickel (Ni) and copper (Cu) [36]. Isomorphous beta stabilizers are highly soluble in titanium [37] compared to eutectoid ones, which are hardly soluble and can form bimetallic bonds [38]. The β phase is stronger and more formable, with improved elasticity, and better matches natural bone mechanical properties, reducing stress shielding, but is harder to process [39]. A comparison between α and β phases can be seen in Table 2.

Apart from isomorphous and eutectoid alloying elements, there are certain elements such as tin (Sn) and hafnium (Hf) that have no effect on either α or β phases [38].

However, titanium alloying metals selection must consider the properties and effects of these elements such as biocompatibility, chemical stability and potential toxic side effects (Mo, V and Al) [40].

Niobium, a ductile transitional metal, with a hardness similar to titanium and Young’s module of 105 GPa, was proposed as a titanium alloying metal for use in medical implant manufacturing because it lacks toxic side effects and is one of the best β phase stabilizers, Ti-Nb alloys displaying superior mechanical properties compared to CpTi (commercially pure titanium) or aluminum vanadium titanium alloys demonstrate increased biocompatibility [41], good corrosion resistance [42] and a low Young’s module, very similar to that of natural bone [43].

The mechanical properties of Ti-Nb implants can be altered to resemble that of the natural bone by varying the wt% of Nb in these alloys; several authors found that the optimal wt% is between 30 and 45 wt% [44,45].

The mean elastic modulus for Ti-Nb alloys is around 40–80 GPa, resembling that of cortical bone and is dependent on the composition and processing techniques, with porous Ti-Nb lowering the elastic modulus by up to 25 GPa, even achieving 3–4 GPa for macroporous Ti-Nb alloys [45,46].

Merhan et al. reported that an elastic module closely resembling that of natural bone (64–66 GPa) was obtained in a Ti-Nb alloy obtained by high-energy ball milling of titanium powder at 900–1000 °C with 34 wt% of Nb [47].

Other authors (Zhang et al.) varied the Nb percentage between 5 wt% to 25 wt% and studied the impact of niobium on β phase formation and mechanical properties of obtained alloys. They found that a 5 wt% of niobium induces sufficient mechanical properties in the obtained alloy; the best characteristics of strength (1000 MPa) and elasticity module (70 GPa) were obtained in an alloy containing 25 wt% [48]. These findings were also confirmed by other authors who found that Ti-Nb for dental implants or hard tissue replacement alloys display an elastic module resembling that of cortical bone [49,50].

As a rule, the elastic module of Ti-Nb alloys decreases and hardness increases with the β phase weight percentage increase [51].

Manufacturing Ti-Nb alloys can use powdered metallurgy technique at 200 rpm followed by controlled pressure and vacuum sintering at temperatures around 1200 °C of titanium hydride (TiH_2_) and niobium powders [52].

Titanium hydride powder can be obtained by the hydrogenation of titanium powder and can be used by controlled dehydrogenation during annealing in an argon-protected atmosphere; the use of titanium hydride has economical advantages (titanium hydride is nine times cheaper than other conventional manufacturing methods) [35]. As the hydrogen to titanium ratio approaches 2, TiH_x_ adopts a modified β phase known as a face-centred cubic structure, known as an δ phase in which the hydrogen atoms fill the tetrahedral sides [53].

Tie_(S)_ + H_2_ -> TiH_2(S)_ (1)

Releasing H_2_ during the heat treatment of this powder can form a protective atmosphere that blocks O_2_ that can react with titanium, thus creating a more compact and more reactive titanium that increases the sintering process [35,54].

Temperature control during the sintering process leads to a more controlled β phase creation, and adding niobium to this process lowers the temperature required for hydrogen release and for β phase titanium formation from δ phase TiH_2_ [55,56].

δTiH_2_ -> δTiH_x_ -> β(Ti)_H_ -> β(Ti)_H_ + α(Ti)_H_ -> αTi (2)

## 3. Titanium–Niobium Alloys Manufacturing Techniques

The most commonly used manufacturing techniques for Ti-Nb alloys are vacuum arc melting, powdered metallurgy, additive manufacturing and hot and cold working [57].

Vacuum arc melting involves melting raw elements under a high vacuum or an inert gas atmosphere using temperature generated by an electric arc, reducing contamination by oxygen and impurities and achieving homogeneity and precise control of the alloy composition [58].

Powdered metallurgy uses metals in a powdered form that are formed and then sintered into the desired shape. It can be applied to both titanium or its alloys and different ceramic materials and can even be used to make new composite materials by mixing alloys and ceramics [59]. Ti-Nb alloys obtained by this process display good physical and mechanical properties and can be shaped in novel forms (for example, porous surface texture or complex geometrical patterns), eliminating uneven or rough textures that traditional castings usually produce [60].

Additive manufacturing, also known as 3D manufacturing, is a combination of computer-assisted design (CAD) and processing methods (computer-assisted manufacturing—CAM) in which computerized model files, through extrusion, sintering, melting, light curing, spraying or other forms of stacking, are used to transform metal or non-metal materials into solid objects [61]. By controlling the parameters of preparation (such as layer thickness and layer speed) and through thorough manufacturing methods, complex parts can be made, such as parts that are not possible with traditional casting methods [60].

## 4. Niobium Implants Biocompatibility

Implant insertion triggers an inflammatory response accompanied by diverse biomolecules such as albumin or other protein depositions on the implant surface, leading to a corrosive medium around the implant [62]. At the same time, there is an increase in oxygen consumption by inflammatory cells, leading to an increase in oxidative stress and also an increase in H_2_O_2_, H_2_O_2_ byproducts such as hypochlorous acid and lactic acid [63,64].

Adding to this already highly oxidant and acidic environment, hypocaloric acid released by osteoclasts produces an acidic environment (pH = 5) at the implant tissue interface that can drop to as low as pH = 2 or 3 in severe cases of peri-implant inflammation, leading to the implant surface corrosion of traditional TiAl6V4 alloys and the formation of highly hydrated porous titanium hydrogen peroxide (TiOOH), negatively impacting osseointegration [65].

Niobium as an alloying metal for titanium implants has very good biocompatibility, good mechanical properties and very good corrosion resistance. Niobium ions are less toxic than vanadium ones and can form potassium niobate (KNbO_3_) after being released from the implant surface by macrophage-induced erosion [66]. Potassium niobate can lower monocyte viability after 72 h and 96 h, and this effect was observed after the in vitro cultivation of Ti-Nb samples covered with the THP-1 cell line (monocyte cellular line) after determining the transcriptomic markers of genes responsible for the growth and inflammatory response of these cells, but this effect is very low and has no significant effect on cellular survivability and function [66]. Chezeau L et al. also investigated the effect of Ti-Nb alloys on SaO_S_-2 (osteoblast cells) and found there is no difference between their viability and growth rate compared to classic Ti-Al6-V4 alloys [66].

Another in vitro study incubated STRO-1A^+^ (human mesenchymal stromal cells) on Ti-Nb implants manufactured by the CLAD technique (laser direct deposition process) and found that the mesenchymal cells have good adhesion, proliferation and viability both on smooth or rough surfaces, with rough surface cultivated cells displaying better results [67].

The corrosion resistance of Ti-Nb alloys was tested in another study using the open circuit potential technique (OCP) after immersing the implant samples in highly fluorinated, nonfluorinated solutions and in acidic saliva and found that compared to traditional titanium implant alloys, niobium alloys significantly increase corrosion resistance [68]. The same study determined the alkaline phosphatase activity (ALP) of L929 (murine fibroblast cells) and MG-63 (human osteoblast-like cells) and found there is no significant modification in ALP expression on Ti-Nb implants versus traditional titanium alloys but there is better adhesion and a better spreading of these cells on Ti-Nb compared with titanium alloys [68].

ALP is an important marker in the initial phases of osteoblast differentiation and is a very good indicator of osteogenic activity [69]. ALP activity and cellular viability were assessed in another in vitro study that focused on the influence of surface porosity and roughness of Ti-35Nb implants and their influence on osteointegration and found that rougher and denser surfaces induce better adhesion and proliferation compared to classic titanium implant alloys [70].

The influence of the surface characteristics of Ti-Nb implants was evaluated in another in vitro study in which a 40% and a 70% porosity surface Ti-25Nb were obtained by titanium–niobium powder sintering in an argon protective atmosphere and at a high temperature after previously being mixed with polyurethane foam, thus varying alloy porosity [71]. This study confirmed that porous surfaces induce better growth and that using the proposed manufacturing technique produced better porosity compared to traditional acid etching [71,72].

Better cell adhesion and growth on porous surfaces can be explained by the close resemblance of these rough surfaces to the structure of the extracellular matrix (ECM), modulating the cytoskeleton and cytoplasmic protein filament network development in cells that adhere to these surfaces [73]. Jian Xu et al., after assessing the cell viability and adhesion on rough surfaces, evaluated IL-6 levels expressed by rabbit bone marrow mesenchymal stem cells cultivated on 40% and 70% roughness Ti-25Nb alloys and compared them to the levels from the classic titanium alloy Ti-Al6-V4; they found that cells cultivated on Ti-Al6-V4 expressed higher IL-6 levels and that there is no difference in IL-6 levels between 40% and 70% roughness Ti-25Nb alloys and concluded that niobium alloys trigger a much lower immune response [71].

The immune response towards Ti-Nb alloys was also studied by J. P. Luo et al., using several inflammatory markers such as TNFα, IL-10, IL-6 and IL-1β expressed by L929 cells cultivated on an electropolished titanium–niobium alloy and compared with 3D-printed alloys. This study found better cellular viability for electropolished alloys compared to the 3D-printed ones and inflammatory cytokine release in both study groups but with lower levels for the electropolished group [74,75]. A higher level of IL-10, a healing process-inducing cytokine was found in later stages in the electropolished titanium–niobium alloy group [75].

A wider spectrum of inflammatory and collagen biomarkers was studied by Aude Falanga et al., including IL-6, IL-8, macrophage inflammatory protein (MIP), vascular epithelial growth factor (VEGF) and matrix metalloproteinases 1 (MMP-1) and collagen expressed by human fibroblasts cultivated on Ti-40Nb alloys. The results showed elevated IL-6 levels, decreased IL-8 levels, an increase in VEGF and equal levels of MIP for Ti-40Nb alloys, confirming a reduced proinflammatory response triggered by this alloy [74].

VEGF is a survival-promoting factor preventing apoptosis and neovascularization, leading to better cellular survival. Collagen released by human fibroblasts exposed to Ti-40Nb alloy is a type III collagen (more elastic), which is first released and then transformed into type I collagen [74].

Another in vitro study used SCP-1 (human mesenchymal cells), SaO_S_-2 and THP-2 cells cultivated on a Ti-Nb alloy treated with a radio frequency O_2_ glow discharge plasma treatment to increase the surface wettability properties and showed better cellular attachment for SCP-1 cells and increased mitochondrial activity (verified by resazurin to resorufin conversion and protein content analysis) for Ti-Nb alloys [20].

Another surface treatment for Ti-Nb implants that was proposed in an in vitro study in order to increase even further the corrosion resistance is nitriding, in which a layer of nitrogen-enriched titanium (Ti_2_N/TiN) is deposited on the surface of a Ti-27Nb alloy. SaO_S_-2, L929 and KB (epithelial cells) were cultivated on the surface of this modified alloy, and the results showed a marginal increase in cell viability compared to traditional titanium alloys, indicating niobium has a good biocompatibility and that nitriding does not interfere with this ability [76]. The nitriding surface treatment leads to better corrosion resistance and a higher superficial hardness [76]. These data on Ti-Nb in vitro biocompatibility are summarized in Table 3.

A novel way of increasing the corrosion resistance of titanium implants is by coating the surface with a polyethylene glycol (PEG) layer doped with Betamethasone (BET), a widely used anti-inflammatory drug, thus achieving reduced inflammation reaction after the implantation triggered by corrosion byproducts [77].

To our knowledge, there are no in vivo studies regarding Ti-Nb alloys’ biocompatibility. The only existing studies used only alloyed niobium either with titanium, tantalum or silver.

Chen et al. investigated the biocompatibility of a TiTaNb coating on Ti-6Al-4V on an animal model using tibial implants, evaluating them using X-rays at 3, 6 and 9 weeks, micro-CT scanning and BMP-2 (bone morphogenetic protein 2) dosing and found that this coating has promising potential due to the Nb_2_O_5_ layer inducing a better osteointegration when compared to normal commercial Ti-6Al-4V implants [78].

Wan et al. developed a niobium silver alloy using 5% wt silver and evaluated its biocompatibility in both in vivo and in vitro studies using animal models and MC3T3 cells (rat osteoblast precursor cells) and found that the Nb-5Ag alloy produces a layer of Nb-OH, inducing apatite nucleation formation and thus promoting osteointegration alongside silver-induced antibacterial properties [79]. 

A Further increase in Ti-Nb alloys’ biocompatibility and osteointegration could be achieved by the addition of metal oxide nanoparticles to the implant surface after anodic oxidation, especially manganese oxide (MnO), leading to the formation of MnO nanotubes after thermal decomposition and then deposition onto an anodized titanium surface [80]. 

We can conclude that niobium as an alloying element for titanium displays good or even better biocompatibility compared to traditional titanium alloys such as Ti-Al6-V4 while decreasing the elastic modulus of this material. It also lacks the side effects of traditional alloying elements such as aluminum and vanadium.

All these findings mainly come from in vitro studies or animal models and require further investigation using more biomarkers and cells to further describe the interactions of niobium with human cells before human trials can be performed.

## 5. Calcium Phosphate-Coated Implants

Titanium and its alloys have ideal mechanical and biological properties but it lacks the ability to establish a chemical link with bone tissue [81].

Enhancing the implant surface properties aims to increase biocompatibility and osteoconductivity since this is the first part of the implant that comes in direct contact with the host [82,83].

Calcium phosphate-based coating materials are composed of naturally occurring ions such as calcium and phosphate, lack any inflammatory or immunogenicity properties and display osteoconductive properties [84] and a chemical composition that is the most similar to that of the human bone, making it the most suitable material for artificial bone manufacturing, various implants and for coating dental implants, thus combining the mechanical properties of metal implants with bioactive properties [85].

The most used calcium phosphate for coating deposition in dentistry is hydroxyapatite (HAp), with the aim of reducing healing time and creating a better osteointegration [86].

HAp coatings must be biocompatible, bioactive and osteoinductive [87]. Furthermore, according to ISO standards, HAp coatings must display a predominant crystalline structure, an amorphic phase, controlled dissolution, strong adhesion to the coated substrate and a similar chemical composition to that of natural bone [87]. Antimicrobial properties are also present after incorporation into the HAp layer of metals like zinc, manganese, silver or copper, enhancing infection resistance through reactive oxygen species (ROS) generation without compromising osteointegration [88,89].

Hap-coated implants display excellent biocompatibility, good osteoconductive behaviour and after ionic substitutions on the HAp layer, good osteoinductive and antibiotic properties [87].

These advantages for Hap-coated implants make them an invaluable option in cases where primary stability and good initial connection between the implant and natural bone are required, such as type IV bone placement, implantation in fresh extraction sites or insertion in recently grafted sites. Another reason for the use of HA-coated implants is in cases of patients suffering from metal allergies [90].

## 6. Hydroxyapatite Deposition Techniques

Calcium triphosphate represents the starting point for most HAp manufacturing techniques since it transforms into crystalline HAp in the presence of water [6].

2H_2_O + 4Ca_3_(PO_4_)_2_ -> Ca_10_(PO_4_)_6_OH_2_ + 2Ca^2+^ + 2HPO_4_^3−^ (3)

The main techniques for HAp layer deposition on various substrates such as dental implants can be divided into physical and wet techniques [91].

Physical deposition techniques are further divided into the plasma spraying method (in which plasma is used to melt and propel particles onto a substrate) [92], RF magnetron sputtering (coating material is sputtered in a vacuum from a target onto a substrate surface using radio frequency energy) [93,94], microarc oxidation (high energy discharges creates a coating on substrate surface submerged into an electrolytic solution) [95], pulsed laser deposition (coating material is vaporized onto the substrate surface using a high power laser) [96] and the ion beam coating method—IBAD (uses focused ion beams to dislodge coating atoms and deposit them on the coated surface) [97].

Wet deposition techniques encompass the electrochemical deposition technique (involving an electrochemical process in a liquid solution) [98], biomimetic deposition technique (mimicking naturally occurring processes in an aqueous solution) [99] and sol–gel technique (uses a solution to create a gel that deposits material on a substrate) [100]. The main physical and wet deposition techniques are listed in Table 4.

The key advantages of physical deposition techniques are thicker coatings, averaging between 50 and 200 µm, strong adhesion, reduced deposition time and the possibility of large industrial applications while the disadvantages are the thermal degradation of the coating compound, uneven surface roughness in some cases and high cost [101,102]. Compared to the previous coating techniques, wet deposition techniques, in general, have a much lower operating temperature, leading to better chemical control and stability of the coating material; these techniques produce even coatings, even with complex substrate geometries, at a lower cost but suffer from a reduced coating thickness (usually between 1 and 10 µm), have lower adhesion and are much slower [34,103,104]. The main differences between physical and wet deposition techniques are summarized in Table 5.

In this review, we concentrate on the physical coating techniques since they are the most widely used methods for titanium surface depositions. Plasma spraying, magnetron sputtering, ion beam deposition and pulsed laser deposition techniques will be discussed in more detail.

## 7. Plasma Spraying

Developed during the 1980s and 1990s, plasma spraying (PS), a method of coating surfaces with various materials, is one of the most used methods for HAp deposition onto metallic structures such as dental or orthopedic implants [105].

The main advantages of this method are a high deposition rate, coating thickness varying between 50 and 200 μm, low cost, good surface characteristics of the deposited layer both in terms of surface roughness and porosity, mainly crystalline coatings obtained and very good adhesion strength of the coated layer to the substrate ranging between 15 and 20 MPa [106,107,108].

The limitations of PS arise from the high temperatures involved that can induce thermal decomposition of the deposited material and cracking due to rapid cooling [109]. Poor control of physical coating parameters such as deposited layer thickness variations stems from being a line-of-sight method [108,110]. It also suffers from poor chemical control of deposition material due to the mixing of the predominant crystalline phase with various regions of the amorphous phase, leading to coating biodegradation [106]. The advantages and disadvantages of this method are summarized in Table 6.

## 8. Plasma Spraying Working Principle

There are several PS deposition techniques such as atmospheric plasma spray, vacuum plasma spray, suspension plasma spray, liquid plasma spray, high-velocity suspension flame spray, high-velocity oxy-fuel, gas tunnel spray and cold plasma spray [106,111].

The working principle of PS involves coating the application of various materials upon the substrate using a high-temperature plasma jet of a mainly argon or hydrogen gas mixture that flows through an electrical arc between an anode and cathode (a plasma torch) reaching around 10,000 °C. This plasma jet melts the coating particles that are introduced into the jet, propelling them at high velocities toward the substrate, where they are rapidly cooled and solidified into a dense and well-bonded coating layer [112]. Powdered PS either in vacuum or at atmospheric pressure is the most used method for HAp coatings on titanium implants [106].

Controlling deposition parameters such as particle velocity, plasma jet temperature, distance to the substrate and atmospheric conditions (vacuum or normal pressure) results in fine control over the deposited layer thickness and properties [113].

## 9. Pulsed Laser Deposition

Pulsed laser deposition (PLD) is a vapour deposition technique that uses a laser source to irradiate the deposition material placed on a fixed plate, creating a gaseous cloud of various particles such as molecules, molecular clusters and various-sized droplets along with atoms, ions and electrons that expand in the vacuum chamber or in a gaseous atmosphere and are deposited onto a rotating substrate holder placed at a distance between 2 and 10 cm, resulting in coating thicknesses from a few nanometers to 1–5 μm [106]. The laser source for HAp coating is usually a KrF excimer with a wavelength of 248 nm, a Nd:YAG source with a 355 nm wavelength or an ArF source with a 195 nm wavelength [114,115].

The biggest advantage of this coating method is the retained stoichiometry of the deposited material due to the high ablation rate causing a simultaneous evaporation [116,117].

Using laser radiation allows for precise temperature control, which, for this method, ranges between 350 and 600 °C, allowing for greater variability in the surface roughness and porosity of the deposition layer, resulting in a highly crystalline structure and reducing the need for post-deposition annealing [118]. This technique can be used even for surface modifications such as modifying the substrate surface microstructure [119].

The main disadvantage of PLD is caused by low-velocity coating particles that cause splashing onto the substrate surface resulting in rough uneven surfaces and coating thickness variations [120]. Another particularity of this technique is that laser interaction with HAp molecules leads to HAp decomposition into various calcium- and phosphate-based compounds like Ca_4_P_2_O_9_, Ca_3_(PO_4_)_2_, CaO, P_2_O_5_ and H_2_O [121]. The advantages and disadvantages of this method can be summarized in Table 7.

## 10. Ion Beam-Assisted Deposition

Ion beam-assisted deposition (IBAD) is a combination of ion bombardment and thin-film deposition such as sputtering or evaporation processes in which an ion beam vaporizes the target molecules, producing a cloud of particles and depositing it on the surface of the substrate that is simultaneously irradiated by high energy ions such as Ar or oxygen produced by a plasma torch [122]. This method produces coating varying from angstroms to micrometres, usually in the 2–4 μm range [123]. This technique uses a low temperature and therefore is suitable for deposition onto thermal-sensitive substrates such as polymers; coatings obtained by this method have improved adhesion, high density and low porosity compared to other deposition techniques but suffer from a low deposition rate and contain almost exclusively the amorphous phase HAp [124,125,126]. High substrate adhesion achieved by IBAD can be explained by the chemical interaction forming on the substrate surface as a consequence of ion bombardment creating an intermixing layer [106]. The advantages and disadvantages of this method are summarized in Table 8.

## 11. RF Magnetron Sputtering

Among HAp deposition techniques, RF magnetron sputtering, a variation in the physical vapour deposition process, has a series of advantages such as high efficiency; controlled deposition rate, allowing for uniform layers with good substrate adhesion; low working temperatures, allowing ideal for thermos-sensible substrate depositions; and lastly the possibility of process automatization [127,128,129,130].

There are also a few disadvantages of using this technique, such as being a line-of sight technique, high cost, low deposition rate, low cohesion HAp layer, amorphous coatings, residual stress and decreased surface roughness [85]. The HAp coating deposited through this method is more amorphous and thus not that stable compared to the ideal crystalline form that mimics the structure of natural bone more closely and requires a heat treatment step after deposition [131]. The average layer thickness is around only 5 μm and requires specialized equipment and vacuum conditions and may not be suitable for applications where thicker layers are needed [89]. The poor mechanical properties of HAp coatings deposited through this method can be caused by delamination due to a lower coating cohesion and through the residual stress being induced [88,89]. The advantages and disadvantages of this method can be summarized in Table 9.

## 12. RF Magnetron Sputtering Working Principle

Rf magnetron sputtering uses high-energy ions from noble gasses such as helium or argon to bombard a surface target made from the coating material. These ions transfer their energy to the coating material atoms, making them vibrate and crash into the surrounding atoms, causing a cascade reaction that eventually ends when the coating atoms break loose from the target surface after enough energy has been transferred and sputter toward the substrate surface where they are deposited [85].

The energy used to accelerate the high-energy ions could come from a direct current or a radio frequency, with radio frequency magnetrons having the advantage of being able to deposit atoms from materials that have poor electric current conductivity or even those that are insulating [132].

Coating layers’ quality and integrity depend on a series of factors such as discharge power, gas flow rate, gas pressure, substrate temperature, post-heat treatment, time and negative substrate bias [106].

A succinct comparison between physical deposition techniques can be found in Table 10.

## 13. HAp Coating Deposited Through Physical Deposition Techniques Characteristics

The ideal ratio of bone calcium to phosphate, 1.67, is achieved by all the physical deposition methods [133], with coatings having a homogenous distribution of Ca and P and reduced undesirable second phases [134]. Slight variations from the ideal 1.67 Ca:P ratio can occur with the formation of Ca_3_(PO_4_)_2_ and CaO for RFm sputtering because not all components of the target material such as oxygen, HO^−^ and hydrogen can be fully transported onto the substrate surface [107,135]. Also, the unequal deposition of Ca and P is another problem, with volatile P_2_O_5_ being replaced with other ions in the coating structure or escaping the reaction chamber in the case of PLD [107,121].

The high temperature developed by the plasma plume influences Ca:P stoichiometry through coating decomposition, leading to an uneven ratio, with coating thickness ranging between 1.5 and 2.2 [136,137]. PS using water suppresses the dihydroxylation induced by the plasma jet that leads to the formation of oxy-apatite and oxy-hydroxyapatite, thus reducing the structural weakness these two forms of apatite induce [138,139].

Further increasing the ideal ratio of Ca:P up to 2.5 was found by Ergun et al. to stimulate osteoblast adhesion, increasing osteointegration [140]. RFm sputtering and IBAD usually produce amorphous phase HAp coatings containing various calcium phosphate compounds (Ca_2_P_2_O_7_, Ca_4_O(PO_4_)_2_, β-TCP, CaHPO_4_, β-CaP_2_O_6_) requiring post-deposition annealing usually at 500–600 °C, higher temperature post-treatment leading to crack and delamination due to recrystallization and HAp volume modification inducing mechanical stress [106,141].

The adhesion of HAp coatings produced by each deposition method is good and can be increased by annealing, using substrate surface modifications such as grit blasting with Al_2_O_3_ or by using intermediate layers (such as TiO_2_ and TiN) that reduce the thermal coefficient difference and increase heat conductivity [142,143,144,145]. Intermediate layers will be discussed further in this review. The best HAp adhesion is obtained after RFm sputtering while the worst adhesion strength is achieved by PLD [120].

The HAp coating hardness is dependent on porosity levels and amorphous and crystalline zone distribution [121,146]. Residual thermal stress during the deposition phase followed by rapid cooling leads to coating deformation and reduced hardness [147]. HAp hardness influences coating strength, surface microcavity stress resistance and abrasion resistance [148,149].

Biocompatibility is not a singular process but an association of processes like osteoconduction, osteoinduction and osteointegration. HAp biocompatibility is related to the ions and particulates released from the coating surface interacting with the host tissues.

Calcium phosphate coating solubility both in vivo and in vitro differs according to the chemical nature of the formed compound, ranging from the lowest to the highest as follows: CaO > α-TCP(α-tricalcium phosphate) > β-TCP > ACP > TTCP (tetra calcium phosphate) > OHAp > HA [107].

CaP coatings enhance cellular adhesion, proliferation and differentiation, and in vitro studies show that cells cultivated on HAp display increased protein and extracellular matrix synthesis; elevated alkaline phosphatase (ALP) expression, indicating osteoblastic differentiation; and elevated osteocalcin production, indicating bone apposition activity [106,150].

The surface roughness of HAp coatings can vary according to the technique used, creating a microtopography resembling the natural surface of bone; this leads to cellular adhesion and promotes the release of growth factors, ALP and an extracellular matrix [101,151,152]. This effect is more pronounced in HAp deposited through RFm sputtering [153].

The HAp thickness influences residual stress, leading to coating dissolution and microcracking, which reduce substrate adhesion and compromise long-term durability and can be controlled by post-deposition heat treatments [154]. The coating thickness also influences the surface morphology; thicker HAp layers have a decreased roughness due to the increase in the deposition time, leading to better particle cohesion and growth [154]. Rougher surfaces translate into better cellular adhesion, enhanced surface protein absorption and lower proinflammatory factors such as TNF- α. Nuswantoro et al. reported that a thickness of 70–90 µm is ideal for long-term osteointegration [101,155]. At first, thicker layers also promote better stability and lead to an increased release of proinflammatory cytokines (IL-1, PGE-2), triggering increased osteoclast activity and potentially inducing bacterial adhesion, leading to marginal bone loss and peri-implantitis [155,156]. 

HAp coatings, particularly octacalcium phosphate ones, can greatly enhance the corrosion resistance of substrate materials, leading to enhanced biocompatibility and osteointegration by further limiting corrosion byproducts’ interaction with the surrounding bone [157].

## 14. HAp Composite Coatings

In order to improve the HAp coating properties deposited through this method, a second phase consisting of various elements can be incorporated by either using two different targets at the same time (co-sputtering) or by sputtering each phase in a sequence, resulting in a composite layer. The first method can be used to sputter oxides and nitrides while the second one is used when HAp is doped with metallic elements such as titanium, silicon or magnesium [158,159,160]. A short comparison between several composite coatings can be found in Table 11.

According to Safavi et al., the HAp phase’s reinforcement elements could be magnesium (Mg), silver (Ag), titanium(Ti), silicon (Si), silicon carbide (SiC), zirconium oxide (ZrO_2_), zirconium nitride (ZrN) and yttrium stabilized zirconia (YSZ) [85].

The addition of magnesium increases mechanical stress resistance by lowering the elastic modulus and enhancing the coating layer toughness [161]. In low quantities, Mg^2+^ is slowly released from the HAp-Mg coating and increases cellular adhesion and proliferation, thus increasing biocompatibility [162]. However, in high concentrations, Mg^2+^ can disrupt the formation of the calcium phosphate matrix [163]. Another effect of the addition of Mg is modifying corrosion resistance. In low concentrations, Mg ions increase corrosion resistance but, at higher levels, they increase porosity and surface roughness, leading to a decrease in corrosion resistance [162].

Silver ions are added for increased antibacterial properties [164]. After the release from the HAp-Ag coating, silver ions diffuse through the bacterial wall where they bind with phospholipids and to -SH radicals of various proteins, disrupting normal membrane transport mechanisms, increasing permeability and leading to bacterial death [165]. It can also substitute calcium ions in the HAp matrix, thus controlling the dissolution [166].

Titanium can be added to increase the bonding strength of the HAp coating with a titanium substrate and to further increase the inert characteristics of titanium since both are chemically similar [161,167].

The addition of Si to HAp stimulates extracellular matrix deposition, which in turn increases the bone regeneration rate [168,169]. Increased cellular absorption onto the HAp-Si coating leads to enhanced interactions between Si and cellular proteins, especially integrin receptors, by activating them and increasing biocompatibility [170]. Si also decreases mechanical resistance by increasing the amorphous phase [85].

Silicon carbide ceramics increase corrosion resistance and mechanical properties [169].

ZrO_2_, ZrN and YSZ additions to HAp improve mechanical properties such as hardness, wear and abrasion resistance and bonding strength alongside the other properties of zirconia such as bio- and chemical inertness and biocompatibility [159,171,172].

## 15. HAp Multilayer or Intermediate Layer Coatings

The difference between the thermal expansion coefficient of titanium and HAp is one of the biggest occurring problems when this type of coating is applied and leads to negative impacts on the mechanical and biological properties of Hap-covered implant surfaces [173]. Applying intermediate layers can overcome the coefficient mismatch issue and improve the mechanical and biological properties of Hap-coated implants by reducing Al, V or Ni release [174,175].

Several coatings are proposed for multilayer coatings such as TiN, YSZ, TiO_2_, Al_2_O_3_, SiC or NbC (niobium carbide) [85,176].

There are several advantages to using intermediate layers such as reducing the need for post-heat treatments that are necessary for reducing the amorphous phase of CaP depositions, thus reducing the dissolution rate of HAp [177]. Another benefit is the heightened adhesion obtained by spraying an intermediate layer of TiN [178,179].

Corrosion resistance is also increased through intermediate or multilayer applications due to the formation of oxide layers (TiO_2_ or N_2_O_5_), reducing porosity levels and increasing adhesion between deposited layers [179,180].

Another consequence of interlayer deposition is the enhanced biocompatibility occurring from the increased surface roughness achieved by this process that enhances surface wettability, leading to better cellular colonization [176].

## 16. Conclusions

Ti-Nb implants are becoming an alternative to cpTi or TiAl6V4 alloys in implant manufacturing due to their improved mechanical characteristics such as Young’s modulus. They also exhibit good in vitro biocompatibility but there is a lack of standardization in their composition and manufacturing techniques. There is also a lack of in vivo studies for these alloys, prompting further research to test their biocompatibility in both in vitro and in vivo scenarios. In contrast, HAp is a widely used coating material for dental and other surgical implants with well-documented properties. In this paper, we reviewed some of the techniques used for HAp coating (physical deposition techniques) and some HAp coating properties that make this type of surface modification an intriguing proposal for Ti-Nb coatings and complement the superior mechanical properties of Ti-Nb and the excellent biocompatibility and osteoinductive properties of HAp. To our knowledge, there are no in vivo studies performed on Hap-covered pure Ti-Nb alloys, which represents a strong argument for further studies combining in vitro research in which new cell lines, new biomarkers (for oxidative stress, extracellular matrix remodelling, inflammation, bone deposition and resorption) and signalling pathways (Akm-Tor) are investigated and hopefully translated into various animal model studies to verify in vitro findings. This would provide further data that could be translated and verified in human trials regarding the clinical usefulness of Ti-Nb and Ti-Nb in HAp-coated implants both for dental and orthopedic applications. 

## Figures and Tables

**Table 1:** Elastic modulus comparison.

	Cortical Bone	Cancellous Bone	Titanium Implants	Ti-Nb
Elastic modulus	10–20 GPa	1.5–2.5 GPa	110 GPa	40–80 GPa

**Table 2:** Comparison between α and β phase titanium alloys.

	α Phase	β Phase
Advantages	Mechanical resistance High ductility Corrosion resistance	Stronger More formable Improved elasticity Reduced stress shielding
Disadvantages	High stiffness Stress shielding	Harder processing

**Table 3:** Niobium biocompatibility in vitro studies.

	Authors	Title	Evaluated Parameters	Observations	Conclusions
1.	Falanga et al. [74]	Niobium-treated titanium implants with improved cellular and molecular activities at the tissue–implant interface	IL-6 IL-8, MIP VEGF MMP-1 collagen	Human fibroblasts cultivated on Ti-40Nb alloy	+IL-6 −IL-8 +VEGF =Levels of MIP for Ti-40Nb alloy −Reduced proinflammatory responses for Ti-Nb alloys !!!
2.	Xu et al. [71]	Potential use of porous titanium–niobium alloy in orthopedic implants: preparation and experimental study of its biocompatibility in vitro.	IL-6	Cells used in the study: rabbit bone marrow mesenchymal stem cells cultivated on 40% and 70% roughness Ti-25Nb alloy compared to classic titanium alloy Ti-Al6-V4	+IL-6 levels in cells cultivated on Ti-Al6-V4 than on Ti-25Nb alloy =IL-6 levels between 40% and 70% roughness Ti-25Nb alloy
3.	Luo et al. [75]	Electropolishing influence on biocompatibility of additively manufactured Ti-Nb-Ta-Zr: in vivo and in vitro.	TNF-α, Il-6, Il-1β Il-10	L929 cells cultivated on an electropolished Ti-Nb alloy and compared with 3D-printed titanium alloys	+Better cellular viability for electropolished Ti-Nb alloys compared to the 3D printed alloys −Lower levels of inflammatory cytokines −Level of IL-10 in later stages in the electropolished Ti-Nb alloy group
4.	Fischer et al. [67]	Synthesis and characterization of Ti-27.5Nb alloy made by CLAD^®^ additive manufacturing process for biomedical applications	STRO-1A^+^	Human mesenchymal stromal cells	Mesenchymal cells have good adhesion, proliferation and viability
5.	Chézeau et al. [66]	In Vitro Molecular Study of Titanium–Niobium Alloy Biocompatibility	Ti-Nb samples	THP-1	No significant effect on cellular survivability and function
6.	de Andrade et al. [70]	Titanium-35 niobium alloy as a potential material for biomedical implants: In vitro study	The influence of surface porosity and roughness of Ti-35Nb	Rougher and denser surfaces	Better adhesion and proliferation
7.	Khrunyk et al. [20]	Synthesis and Characterization of a Novel Biocompatible Alloy, Ti-Nb-Zr-Ta-Sn.	SaO_S_-2 and THP-2 cells	Human mesenchymal cells	Increased mitochondrial activity
8.	Bédouin et al. [76]	Enhancement of the biocompatibility by surface nitriding of a low-modulus titanium alloy for dental implant applications.	SaO_S_-2 L929 KB	Ti_2_N/TiN	Cell viability Good biocompatibility Better corrosion resistance Higher superficial hardness

**Table 4:** Hydroxyapatite coating techniques.

Physical Coating Techniques	Wet Coating Techniques
Plasma spraying	Electrochemical deposition
RF Magnetron sputtering	Biomimetic deposition
Ion beam coating	Sol–gel deposition
Pulsed laser deposition	
Microarc oxidation	

**Table 5:** Main differences between physical and wet deposition techniques.

	Physical Deposition	Wet Deposition
**Coating thickness**	50–200 µm	1–10 µm
**Adhesion strength**	High	Moderate
**Substrate topography**	Simple geometries	Complex geometries
**Coasting stability**	Moderate	High
**Durability**	High	Low
**Cost**	High	Low

**Table 6:** Plasma spray HAp technique: advantages and disadvantages.

Plasma Spray HAp Coating Advantages	Plasma Spray HAp Coating Disadvantages
High deposition rate	High-temperature technique
Thick coatings	Coating material thermal decomposition
Low cost	Coating cracks
Good surface roughness	Variable coating thickness
Good surface porosity	Coating biodegradation
Good crystallinity	
Very good substrate adhesion	

**Table 7:** PLD HAp coating: advantages and disadvantages.

PLD HAp Coating Advantages	PLD HAp Coating Disadvantages
Retained stoichiometry of coating material	High degree of splashing
Good layer variability parameters	Decomposition of HAp coating into various calcium- and phosphate-based compounds
High crystalline HAp coating	
Reduced post-deposition annealing	
Surface modification	

**Table 8:** IBAD HAp coating: advantages and disadvantages.

IBAD HAp Coating Advantages	IBAD HAp Coating Disadvantages
Improved adhesion to substrate	Low deposition rate
High density	High percentage of amorphous HAp
Low porosity	Complex equipment
Low-temperature deposition	High cost

**Table 9:** RF magnetron HAp sputtering: advantages and disadvantages.

RF Magnetron HAp Sputtering Advantages	RF Magnetron HAp Sputtering Disadvantages
High efficiency	Line-of-sight technique
Controlled deposition rate	High cost
Uniform deposited layer	Low deposition rate
Good substrate adhesion	Low layer cohesion
Low working temperature	Predominant amorphous HAp
Automation potential	Residual stress
	Decreased surface roughness

**Table 10:** Comparison between the advantages and disadvantages of several physical deposition techniques.

Deposition Technique	Advantages	Disadvantages
**Plasma spray**	High deposition rate Thick coatings Low cost Good surface roughness Good surface porosity Good crystallinity Very good substrate adhesion	High-temperature technique Coating material thermal decomposition Coating cracks Variable coating thickness Coating biodegradation
**Pulsed laser**	Retained stoichiometry of coating material Good layer variability parameters High crystalline HAp coating Reduced post-deposition annealing Surface modification	High degree of splashing Decomposition of HAp coating into various calcium- and phosphate-based compounds
**IBAD**	Improved adhesion to substrate High density Low porosity Low-temperature deposition	Low deposition rate High percentage of amorphous HAp Complex equipment High cost
**RF Magnetron**	High efficiency Controlled deposition rate Uniform deposited layer Good substrate adhesion Low working temperature Automation potential	Line-of-sight technique Low deposition rate Low layer cohesion Predominant amorphous HAp Decreased surface roughness Residual stress High cost

**Table 11:** Actions of HAp composite coating elements.

Composite Coating Element	Actions
Mg	Increases mechanical strength Lowers elastic modulus Enhances toughness Modified corrosion resistance Controls HAp dissolution Increases cellular adhesion and proliferation
Ag	Antibacterial properties Controls dissolution
Ti	Increases bonding strength Increases inert titanium characteristics
Si	Stimulates extracellular matrix deposition Increases cellular absorption Decreases mechanical resistance
SiC	Increases mechanical properties Increases corrosion resistance
ZrO_2_ ZrN YSZ	Improve hardness Improve wear and abrasion resistance Increases bonding strength Increases bio and chemical inertness Increases biocompatibility
